# Multi-Site evaluation of a novel point-of-care 3D printing quality assurance protocol for a material jetting 3D printer

**DOI:** 10.1186/s41205-025-00259-w

**Published:** 2025-03-06

**Authors:** Matthew D. Marquardt, Nicholas Beemster, William Corcuera, Dylan T. Beckler, Kyle VanKoevering, Megan Malara, Teri Snyder, Zachary C. Thumser

**Affiliations:** 1 The Ohio State University College of Medicine, Columbus, Ohio USA; 2 Center for Design and Manufacturing Excellence, The Ohio State University, Columbus, OH USA; 3 Department of Integrated Systems Engineering, The Ohio State University College of Engineering, Columbus, OH USA; 4 Louis Stokes Cleveland Department of Veterans Affairs Medical Center, Cleveland, OH USA; 5 Department of Otolaryngology – Head and Neck Surgery, The Ohio State University, 10701 East Boulevard, Cleveland, OH 44106 USA

**Keywords:** Quality assurance, 3D printing, Dimensional accuracy, Point of care manufacturing, Patient specific modeling

## Abstract

**Background:**

The maturation of 3D printing technologies has opened up a new space for patient advancements in healthcare from trainee education to patient specific medical devices. Point-of-care (POC) manufacturing, where model production is done on-site, includes multiple benefits such as enhanced communication, reduced lead time, and lower costs. However, the small scale of many POC manufacturing operations complicates their ability to establish quality assurance practices. This study presents a novel low-cost quality assurance protocol for POC 3D printing.

**Methods:**

Four hundred specially designed quality assurance cubes were printed across four material jetting printers (J5 Medijet, Stratasys, Eden Prairie, Minnesota, USA) at two large medical centers. Three inner dimension and three outer dimension measurements as well as edge angles were measured for every cube by trained research personnel. The delta and absolute error was calculated for each cube and then compared across variables (axis, material, inner vs. outer dimension, swath and machine/site/personnel) using ANOVA analysis.

**Results:**

Print axis and inner vs. outer dimension of the model produced statistically significant differences in error while there was no statistically significant difference in the error for material, print swath, or machine/site/personnel. For the print axes, the printers produced an average error of 26, 53, and 57 μm and the error at three sigma was found to be 100, 158, and 198 μm for the Z, R, and Theta axes, respectively.

**Conclusion:**

This study demonstrates that this novel protocol is both feasible and reliable for quality assurance in POC 3D printing across multiple sites. This protocol offers an adaptable framework that allows users to tailor the QA process to their specific needs. Through the comprehensive method, users can measure and identify all relevant factors that might introduce error into their printed product and then follow the most critical aspects for their situation across every print. The QA cubes produced via this protocol can provide guidance on print quality and alert users to unsatisfactory machine operation which could cause prints to fall outside of engineering and clinical tolerances.

**Supplementary Information:**

The online version contains supplementary material available at 10.1186/s41205-025-00259-w.

## Introduction

In the past decade, the maturation of 3D printing technology has heralded in a new era in manufacturing. Prices have dropped substantially, enabling various industries to take advantage of 3D printing’s unique flexibility in design and production [1]. In healthcare, 3D printing is revolutionizing patient care and treatment. It has been adopted for a multitude of applications including patient education, surgical planning, surgical guides, custom protheses and more [2–5]. Although these devices can be produced by commercial vendors, on-site 3D printing labs and manufacturing centers are becoming increasingly common, a practice now known as point-of-care (POC) manufacturing [1, 3, 5].

Point-of-care 3D printing leverages enhanced communication between end users, designers, and manufacturers. Proximity and direct communication between physicians and on-site manufacturing engineers allows for rapid production and delivery of models for use in time sensitive clinical or surgical situations [5–7]. In the surgical setting, where most research on 3D printed models has been focused, the use of patient-specific models by surgeons has been shown to improve surgeon effectiveness, reduce surgeon fatigue, reduce operative time, reduce ischemia time, and reduce hospital costs [3, 8–11]. The ability to produce bespoke models and devices on demand promises a future where treatments are more effective by being tailored to the individual needs of patients [1].

Quality assurance (QA) is a crucial process in the production of patient-specific medical models to ensure that segmented anatomy is reproduced without distortion given the high stakes involved in medical settings [2, 12, 13]. While commercial vendors have robust QA processes in place, POC manufacturers historically have not been subject to the same level of scrutiny or regulatory oversight [14]. Despite the importance of QA, the topic has only recently begun to garner attention. A systematic review identified 139 articles addressing quality assurance published between 2017 and 2022, with the highest annual output reaching 28 articles in a single year [13]. Error in the production of models can occur during four main stages of production: image acquisition, segmentation, digital editing, and printing [13]. Studies evaluating printer error are the most common due to high variation in the types of printers and materials used at this step [13]. However, most of these studies assess print error for only a specific anatomical indication such as a femur or a mandible [13, 15–17], and none to the study team’s knowledge have presented a process that can be implemented for every print. Growing adoption and expansion of clinical indicators for the use of patient-specific 3D models necessarily increases the need for the design and validation of a simple and cost-effective QA process [3, 12, 13]. The production of test coupons alongside 3D printed models is a QA concept that has been endorsed by the FDA for additive manufacturing [18]. The idea for a simple solid cube to serve as the test coupon has been previously demonstrated as feasible but has not been integrated into a comprehensive QA protocol [19]. Achieving a simple and low-cost QA process is crucial to establish POC manufacturing in healthcare as a credible source of patient specific 3D models.

Recognizing this need, a novel quality assurance protocol that addresses the gap in standardized QA processes for POC 3D printing is presented and evaluated. While this work uses the Stratasys J5 Medijet printer, a material jetting technology, the principles behind the methods are scalable and generalizable to other 3D printers, addressing a crucial weakness in POC manufacturing for healthcare. It is hypothesized that this protocol is implementable across multiple sites and will provide insights into which aspects of the tested printers have significant effects on the dimensional accuracy of prints.

## Methods

### Printing the quality assurance cubes

A multicenter, multi-printer pilot study of a new manufacturing and quality assurance protocol was conducted during the summer of 2023 at one large academic medical center and one Veteran’s Affairs medical center in the Midwest. The purpose of the pilot study was to validate a quality assurance protocol using the material jetting 3D printers (J5 Medijet, Stratasys, Eden Prairie, Minnesota, USA)—which can be generalized to other printer technologies. No human subjects were involved, removing the need for IRB approval.

Across two sites, four material jetting 3D printers were included in the study (three printers at site 1 and one printer at site 2). Specially designed QA cubes—dimensional drawings found in Supplement 1—were printed by each printer in 20 sets of 5 cubes. The cube was designed with circular cutouts on each face so that both inner and outer dimensions could be measured, enabling us to identify how different geometries might influence error. The three orthogonal faces of the cube were printed with labels which corresponded to operational axes of the printer. In total, 400 cubes were produced across the four printers. Mechanically, the printer uses cylindrical coordinates, so all cube and printer dimensions are expressed as R, Theta, and Z. Theta is the dimension of the rotating, turntable-style build platform which spins at a continuous rate during printing and to which all models are firmly attached during printing. R is the radial motion of the print head assembly which moves all print heads as a single solid unit along a single degree of freedom rail, orthogonal to the direction of rotation. This dimension comprises an inner, middle, and outer “swath”—i.e., area on the build plate/layer—that covers the full print layer in no more than three passes. Finally, Z is the position of the build plate as it is slowly lowered each layer away from the printing plane of the print heads. GrabCAD (Stratasys, Eden Prairie, Minnesota, USA) was used to set up and initiate all prints. To assess the impact of print placement on dimensional accuracy, 10 sets of cubes were printed on the outer swath (i.e., outer circumference) of the build plate and 10 sets of cubes were printed on the inner swath (i.e., inner circumference) of the build plate for each printer. Note, the rotational speed of the Theta axis is held constant during a print; however, the speed is adjusted depending on the swath(s) used to limit the maximum linear rate that the build plate passes the print head assembly. As a result, a print including *both* the outer and inner swath will rotate roughly half as fast as a print using only the inner swath to keep the outer swath’s linear rate within the predetermined limit. For prints using only the outer or the inner swath—which is the case in this protocol—the build platform passes the print heads at the maximum linear rate for whichever swath is being used, representing a “worst case” scenario for print accuracy. QA cubes were always oriented with the black stripe on the orientation plate parallel to the curve of the build plate edges (Supplement 2). Only QA cubes were printed for each print run. All cubes were post-processed according to standard practice, and the orientation plates were discarded.

### Measuring the cubes

Six linear distance measurements were taken from each cube: three outer dimensions, and three inner dimensions. These outer and inner measurements were made with digital calipers, using the outer- and inner-dimension jaws, respectively. All measurements were taken so that the caliper tongs were colinear with the axis of the face being measured, as shown in Supplement 1. Importantly, the label on each face corresponded to the axis which ran perpendicular to that face. For the outer dimensions, the distance between each pair of orthogonal faces were measured and recorded. An inner dimension was also measured at the widest part of the inner circle on the corresponding faces. Like with the outer dimensions, the tongs were aligned to the axis of interest, as indicated by the label printed on the corresponding face, as shown in Supplement 1. Angular accuracy of the faces was also assessed via a pass/fail test where the cube was held tight against a machinist square and a visual inspection was conducted to determine whether any skew was present as shown in Supplement 1. All cubes that were produced passed this final check, so no further analysis of the skew measurement is included here. At site 1, one professionally educated and trained engineer conducted all measurements, and at site 2, one trained undergraduate technician completed all measurements. To reduce the risk of fatigue-induced errors and to ensure accuracy, no more than 20 cubes were measured by a technician during one sitting and no more than 30 cubes could be measured during one workday. The complete work instructions can be found as Supplement 1.

### Analysis

All analysis was conducted in JASP (University of Amsterdam, Amsterdam, Netherlands). Delta and Error were the two main statistical endpoints of this study. Delta was defined as measured dimension minus nominal dimension whereas Error was defined as the absolute value of the measured dimension minus the nominal dimension. Descriptive statistics summarized all delta values, all error values, and error values dependent on the material used.

An ANOVA was conducted for both Delta and Error measurements. This analysis assessed the following variables for differences in outcomes: Axis (Theta vs. R vs. Z), Material (Cyan vs. Magenta vs. Transparent vs. White vs. Yellow), Cube Dimension (Inner dimension vs. Outer dimension), Swath (Inner circumference vs. Outer Circumference) and Machine—which encompasses the cumulative effect of the site, printer, and measurer. The following interactions between the variables were also analyzed for significance: Axis-Cube Dimension; Axis-Swath; Axis-Machine; Material-Machine; Swath-Machine. It is important to note that all materials across all machines were identical except for Transparent, where Site 1 used Ultraclear and Site 2 used Biocompatible Clear. While the two transparent materials have very similar properties, there was the potential for this to impact results.

## Results

**Fig. 1:**
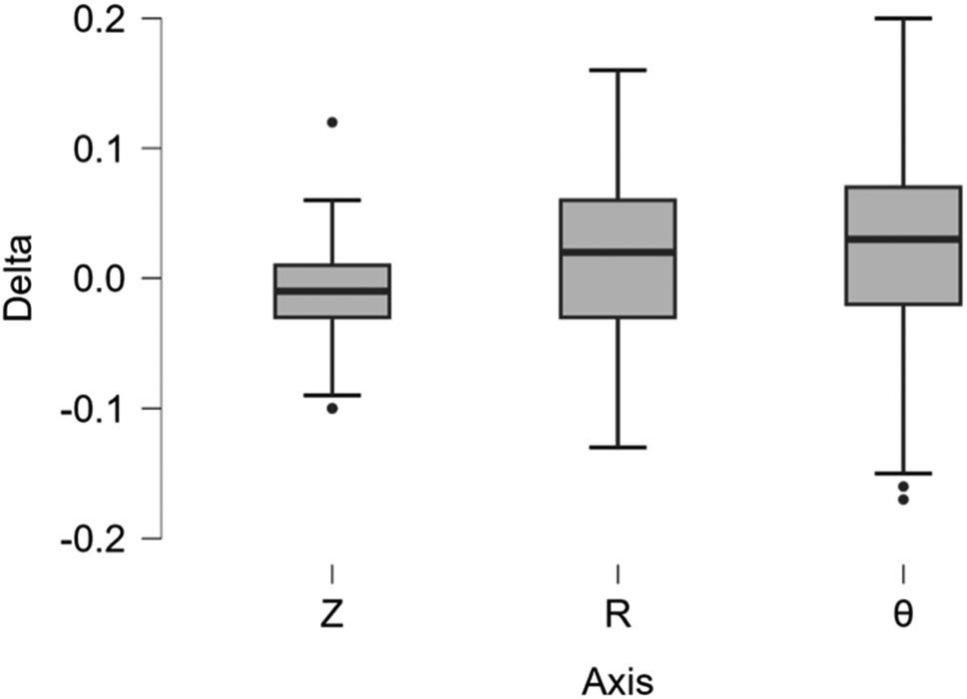
Boxplot of delta measurements for all cubes depending on cube print axis

Table 1 summarizes the delta values for all measurements for each printer axis across Z, R, and Theta. The average deltas were − 11, 17, and 23 μm for Z, R and Theta, respectively. More importantly, the standard deviation of the delta, which demonstrates the variability in accuracy from print to print was 32, 62, and 67 μm for Z, R and Theta, respectively. Figure 1 shows box plots encompassing the variation in the prints from the nominal values. Individual dots represent outliers. There was no statistically significant difference between the axes.

**Table 1:** Descriptive summary of Delta values for all cubes depending on cube print axis

Delta			
Axis	Z	R	θ
Number of Measurements	400	400	400
Mean	-0.011	0.017	0.023
Standard Deviation	0.032	0.062	0.067
-3 σ (sigma)	-0.100	-0.118	-0.158
-2 σ (sigma)	-0.070	-0.090	-0.080
-1 σ (sigma)	-0.020	-0.020	-0.010
+ 1 σ (sigma)	0.010	0.050	0.060
+ 2 σ (sigma)	0.040	0.120	0.130
+ 3 σ (sigma)	0.060	0.158	0.198

Table 2 summarizes the error values for all measurements for each printer axis across Z, R, and Theta. The average error was 26, 53, and 57 μm while the standard deviation was 21, 37, and 41 μm for the Z, R, and Theta axes respectively. Additionally, for medical use, standard quality assurance guidelines call for error at three sigma, which was 100, 158, and 198 μm for the Z, R, and Theta axes, respectively.

**Table 2:** Descriptive summary of error values for all cubes depending on cube print axis

Error			
Axis	Z	R	θ
Number of Measurements	400	400	400
Mean	0.026	0.053	0.057
Standard Deviation	0.021	0.037	0.041
1 σ (sigma)	0.030	0.070	0.070
2 σ (sigma)	0.070	0.120	0.140
3 σ (sigma)	0.100	0.158	0.198

Table 3 presents the ANOVA results for the error analysis with degrees of freedom and p-values reported for single and two variable interactions. The results reveal that among the single variables, Axis and Inner/Outer Cube Dimension had significant results (*P* < 0.05) and for two variable interactions all interactions assessed—Axis-Inner/Outer Cube Dimension, Axis-Build Plate Location/Swath, Axis-Machine, Material-Machine, and Swath-Machine—had significant results (*P* < 0.05). The results for Material and Machine alone were not significant (*P* > 0.05).

**Table 3:** ANOVA results for error measurements based on variable

ANOVA of Error by Variable				
Variable	Sum of Squares	Degrees of Freedom	F	*p*
Axis	0.120	2	58.427	< 0.001
Material	0.009	4	2.301	0.057
Inner vs. Outer Dimension	0.015	1	14.665	< 0.001
Swath	0.003	1	3.077	0.080
Machine	0.007	3	2.245	0.081
Axis-OD/ID	0.058	2	28.456	< 0.001
Axis-Swath	0.014	2	6.844	0.001
Axis-Machine	0.071	6	11.505	< 0.001
Material-Machine	0.023	12	1.834	0.039
Swath-Machine	0.011	3	3.423	0.017
Residuals	1.194	1163	--	--

**Fig. 2:**
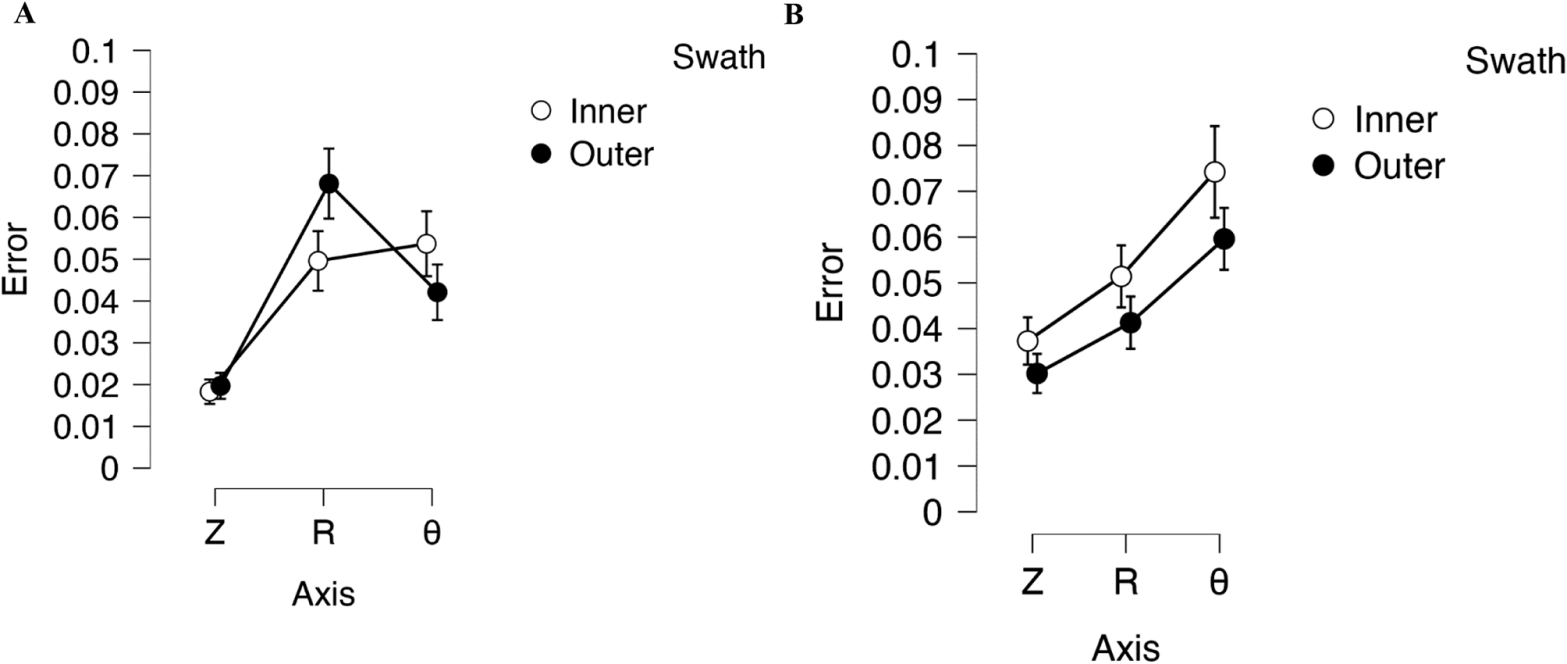
Comparing the error for each cube print axis based on swath/print location for **A**) cube inner dimension and **B**) cube outer dimension

Figure 2 displays the difference in error for the inner dimensions (Fig. 2A) and the outer dimensions (Fig. 2B) of the cube depending on the print axis and the swath—i.e., inner circumference vs. outer circumference—that the cubes were printed on. Outer dimension had a statistically greater magnitude of error for the Z and Theta axes, but not the R axis. There was no statistically significant difference in the error magnitude between the inner and outer swaths except for the R axis on the inner dimension.

**Fig. 3:**
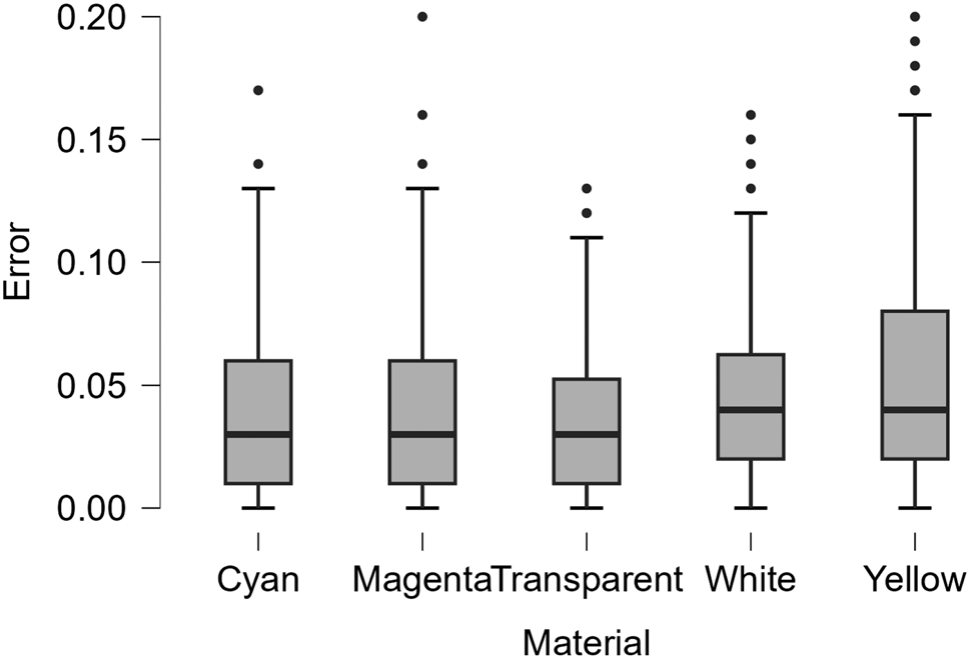
Boxplot comparing the overall error for each cube depending on print material

Table 4 summarizes the error based on print material while Fig. 3 displays boxplots of the error depending on the material and the print axis. Figure 3 provides further evidence that the material used does not influence the accuracy of the print, regardless of the axis dimension. Table 5 demonstrates the differences in error based on material color.

**Table 4:** Descriptive summary of error depending on cube material

Error by Color					
Color	Cyan	Magenta	Transparent	White	Yellow
Number of Measurements	120	114	132	120	114
Mean	0.041	0.043	0.039	0.046	0.058
Standard Deviation	0.036	0.038	0.032	0.046	0.058
+ 1 σ (sigma)	0.050	0.050	0.050	0.059	0.070
+ 2 σ (sigma)	0.111	0.120	0.100	0.110	0.153
+ 3 σ (sigma)	0.159	0.186	0.126	0.156	0.197

**Table 5:** Descriptive summary of error depending on cube material

Error by Color					
Color	Cyan	Magenta	Transparent	White	Yellow
Number of Measurements	120	114	132	120	114
Mean	0.041	0.043	0.039	0.046	0.058
Standard Deviation	0.036	0.038	0.032	0.046	0.058
+ 1 σ (sigma)	0.050	0.050	0.050	0.059	0.070
+ 2 σ (sigma)	0.111	0.120	0.100	0.110	0.153
+ 3 σ (sigma)	0.159	0.186	0.126	0.156	0.197

## Discussion

The use of 3D printing to create patient-specific models is set to continue growing in the future as more institutions adopt the technology and more indications for its use are developed. POC 3D printing—where the model is produced onsite—provides significant benefits through rapid turnaround time and improved communication with the ordering provider [7, 12]. However, there is a need for a simple and low-cost QA process that can be conducted for every model to ensure that models meet the stringent accuracy standards demanded by healthcare [3, 13]. This multicenter, pilot study trialed a novel manufacturing and quality assurance protocol to evaluate the real-world dimensional accuracy of the Stratasys J5 Medijet printer. Through this study the following objectives were achieved: (1) demonstrated a proof of concept for a novel, low-cost QA process across multiple sites and printers, (2) calculated the variation in error for each print axis up to three sigma, and (3) determined which print variables and their interactions produce significantly different magnitudes of error. *Crucially*,* this protocol provides an adaptable framework that enables users to identify and measure the main factors that could influence print accuracy*,* determine which factors are the most pertinent to their situation*,* and then implement a simple process for every print that enables them to continuously assess these factors.* Importantly, while clinical needs inform the requirements for final model accuracy, they do not fully define the QA process tolerances as errors within QA tolerances can indicate machine or print failures even if the errors meet clinical accuracy requirements).

Understanding which print variables produce statistically different magnitudes of error and why, is essential for any QA process to be valuable and actionable. In the following sections, where deviations were occurring in the machines and what might be causing them are highlighted.

### Axis and interactions

The axes produce statistically significant different magnitudes of error as demonstrated by Tables 2 and 3. This result is most likely driven by the independent nature and dramatically different mechanical motions of all three axes, which is supported by the differences in both the Delta (Table 1) and the Error (Table 2) variability where R and Theta produced similar standard deviations whereas the Z axis had a much lower standard deviation. Importantly, the magnitude of this effect is substantial as indicated by the high F value of 58.4, suggesting that the error introduced by the axes is likely the primary contributor to the statistical significance observed in all interactions involving Axis (i.e., Axis-Inner/Outer Cube Dimension, Axis-Build Plate Location/Swath, and Axis-Machine). Additionally, the results confirm the accuracy claims made by the manufacturer as the study team found a 1 sigma error of 70 μm across all axes, which is well within the 150 μm accuracy advertised by Stratasys for this printer [20].

### Cube outer dimension and inner dimension

The inner dimensions and the outer dimensions of the cube also displayed statistically significant differences in error (Table 3), suggesting that print error is different for concave features—the internal features of the QA cubes—than for the external features of the QA cubes. As a result, it is suggested that QA cube inner and outer dimension measurements should be kept as a key step for any generalizations made to this process, with the user setting their own thresholds for acceptable levels of variation. Print axis and build plate location did not influence QA cube inner and outer dimensional accuracy in a significant manner, except for the R axis for the inner dimension (Fig. 2). This variation was only present for the inner swath of a single printer and does not produce a pattern across machines, leading us to believe that it is likely caused by a small number of cubes and that it likely will disappear with additional measurements.

### Material

In theory, there is potential for the material and print color to have an impact on dimensional accuracy (Table 5). However, these results suggest that material does not influence error. Vero cyan, magenta, and yellow all have very similar chemical compositions, so the similarity in their error is not surprising. Importantly, neither transparent nor white materials had significantly different levels of error either, further demonstrating that material does not influence dimensional accuracy. While there is a significant effect observed for the interaction between Material and Machine, this is most likely due to print head health, underscoring the importance of following manufacturer guidelines related to print head maintenance.

### Swath and interaction with machine

Swath/build plate location did not have a significant effect on cube error. This suggests that the positioning of the QA cube on the build plate does not influence error outcomes, allowing users to prioritize their main print objectives and place a QA cube on any suitable location on the build plate. Other quality assurance studies for polyjet printers have also found that print location does not influence accuracy [21]. A significant interaction was found between Swath and Machine, but this is most likely due to noise, rather than an important trend, since a deeper analysis of the data found that this result seems to be driven by one machine.

### Machine

A key outcome of this study is the non-significance of the Machine variable, indicating that variations attributed to the Machine—i.e., the printer, the site, and the measurer combined—do not significantly affect error (Table 3). This finding underscores the robustness and repeatability of this proposed QA protocol, and points to the potential for broader implementation of this protocol without the concern of significant variability introduced by machine, person, and/or site related factors.

### Limitations

This study does have a few limitations that must be noted. The primary limitation is the small sample size; however, the study team sought to reduce the chance for noise to impact the results by following a stringent work order and by printing 100 cubes with each printer. It also must be noted that while the machine, site and person did not have an influence on the error in this study, these factors could easily become a source of error; however, this risk can be minimized through proper training and strictly following this protocol. Another limitation is that the two sites used slightly different versions of transparent (Ultraclear and biocompatible clear); however, this discrepancy did not produce a significant effect on dimensional accuracy. Additionally, this specific protocol is designed for the Stratasys J5 Medijet printer. As a result, this protocol cannot be directly applied to other printers; however, its working principles can be readily generalized to other printers and situations.

### Overall and future work

The findings of this study demonstrate the viability of this proposed protocol as an effective quality assurance process for the J5 Medijet printer and highlight its potential for adoption across various sites and printer models within POC 3D printing settings. The results suggest that dimensional error is driven by the print axis and a model’s geometry, and is not impacted by the site, measurer, machine, location of print on the build plate or the material used. The machine-material interaction additionally emphasizes the importance of following manufacturer guidelines for printer and nozzle maintenance. Given the success of this study, both sites have fully integrated this protocol into their workflows for producing models used in real clinical and surgical situations. It is reasonable to assume that this protocol is directly generalizable to other Stratasys J55 series material jetting printers as the hardware is identical to the Medijet version that was tested. However, the accuracy results might differ, especially if other materials are used. Future work should seek to generalize this protocol to other printer models as well as adding additional cubes and sites to this dataset to further validate the reliability of this process.

## Conclusion

Increasing adoption of 3D printing in the Point-of-Care setting has created a need for a simple and cost-effective quality assurance process that can be implemented with each print. In response to this need, the results of a multisite feasibility trial of a low-cost, novel quality control protocol are presented. While anatomic and clinical requirements are important for determining print accuracy, this QA process enables the identification of machine or print failures even when clinical accuracy standards are met. This protocol provides a simple yet adaptable framework that can inform users about print quality by comprehensively measuring and identifying the most relevant factors that determine print error. Users can then determine which error matters most to their situation and assess the influence of those factors with every print.

## Electronic supplementary material

Below is the link to the electronic supplementary material.

Supplementary Material 1

Supplementary Material 2

## Data Availability

The datasets used and/or analyzed during the current study are available from the corresponding author on reasonable request.
