# Supplementary material for: Multi-Site evaluation of a novel point-of-care 3D printing quality assurance protocol for a material jetting 3D printer
Source: 3D Print Med. 2025 Mar 6;11:10. doi: 10.1186/s41205-025-00259-w (PMC11883906; doi:10.1186/s41205-025-00259-w)

# **DRAFT: Manufacturing and Quality Assurance (QA) Procedure for Creation and Measurement and Standards for using the Stratasys J5 Medijet printer.**

## **Purpose:**

We are undergoing a Quality Study Project to evaluate a novel quality assurance process with cube test prints (FDA coupons).

A total of 100 cubes will be printed in sets of 5 cubes each. Ten (10) prints will have the cubes oriented along the inside of the build plate and the other 10 prints will have the cubes oriented along the outside of the build plate. Run integrity will be maintained for example, (Inner 1-10) and Outer (1-10).

Planning

The document “Cube Study Tracking.xlsx” documents and records the printing and post-processing of the cubes. The document must be updated as soon as a print is started, or post-processing is finished.

## **Printing the Cube**

Anatomy of the Cube

The QA Cube consists of three main components, each consisting of several smaller bodies. (See “QA Cube Standard.pdf” for a 3D image of the full assembly.)

1. The cube – primary component of the QA cube from which all dimensions are measured. See “QA Cube Drawing.doc” for a mechanical drawing with dimensions. The cube body has been pre-configured in “VA Cube Study Print.print” to be clear.
   1. (This must be done manually using Model Settings and Tray Materials if importing from the .3MF files.) All other colors are pre-configured and are checked by the next subcomponent.


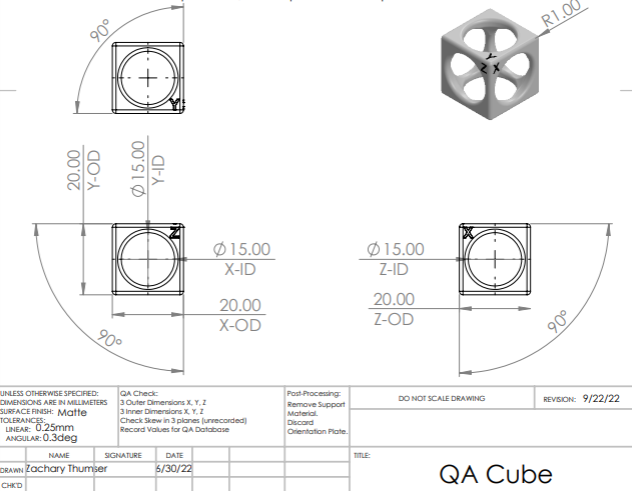


*Figure 1: “QA Cube Drawing.doc” cube dimensions*


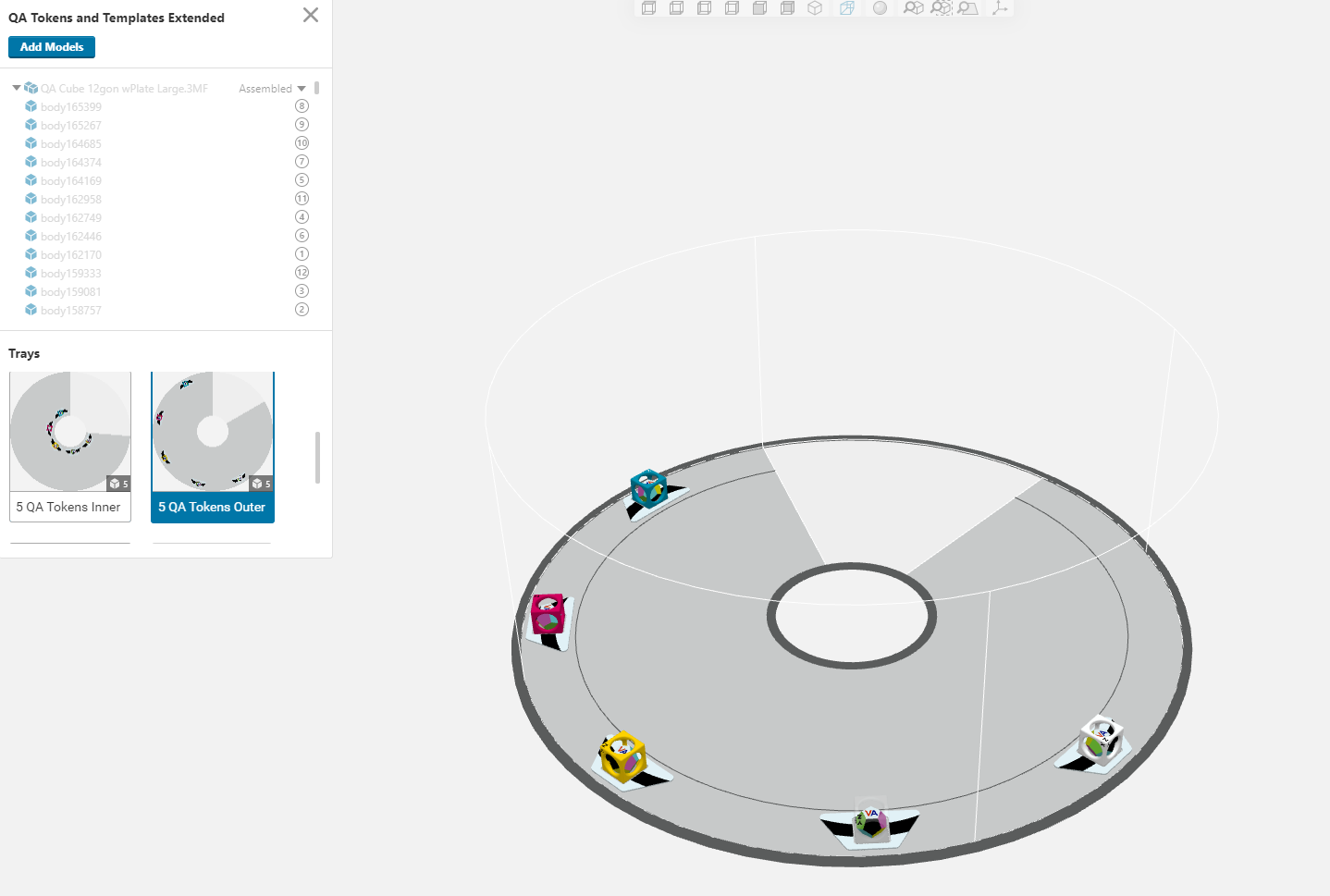


*Figure 2: Opening “VA Cube Study Print.print” in GrabCAD Print*

1. Caged polyhedron – both the standard and large QA cube include a caged dodecahedron. Solid faces are colored cyan, magenta, yellow, black, and green (two of each), with two white faces featuring “VA” printed in red and blue. If model color is not critical, or to save material, the caged polyhedron and “VA” bodies may be omitted without affecting QA validity.


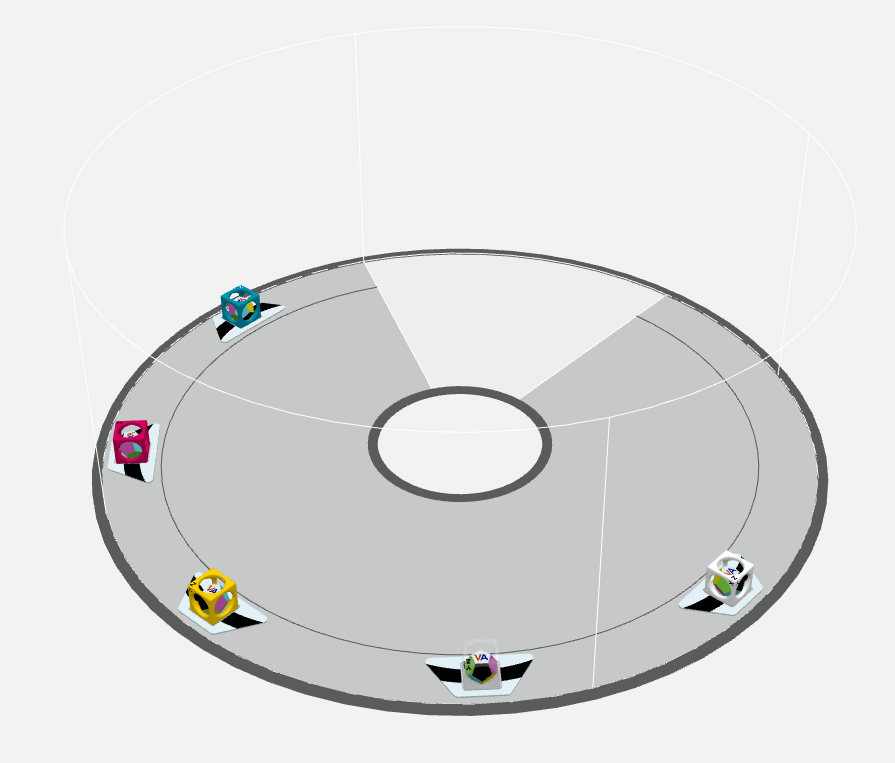


*Figure 3: “5 QA Token Outer” file on GrabCAD plate*


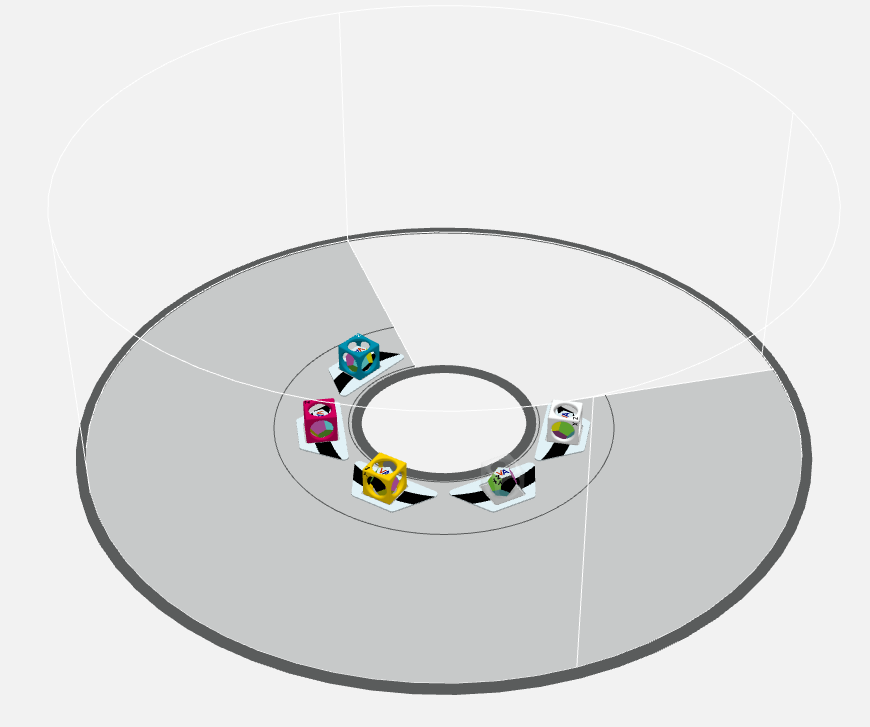


*Figure 4: “5 QA Token Inner” file on GrabCAD plate*

1. *Orientation plate* – to assist in the proper plating of the QA cube. The cube must be placed so that the curved black stripe on the orientation plate curves in the same direction as the rotation of the build plate.

# **GrabCad and Production**

1. Open the following print file, “VA Cube Study Print.print”, in GrabCAD to print the cubes onto the J5.
2. When the print file is open on GrabCAD, ensure that the orientation plates and the prints are identical to Figure 3 for “5 QA Token Outer” and Figure 4 for “5 QA Token Inner”. The orientation of the cubes in the print are fixed, so you should not have to make any alterations to the orientation.
3. Select the print that will be performed (“5 QA Token Outer” or “5 QA Token Inner”).
4. Select the “Stratasys J5 Printer” to send the print to the printer. Click “print” and then select acknowledge.
   1. GrabCAD will ask you to acknowledge that the usual biocompatibility settings are different – do not worry about this message and click “Acknowledge” to move to the next step.
5. Go to the Stratasys J5 Printer and confirm that the print has sent. Once confirmed that the print is ready on the Stratasys J5 Printer, select the “▶” icon.
6. Document the print in the cube production tracking sheet.

# **The Use of the Cube-Procedure:**

Every build plate shall include one QA cube. If all key anatomical features (KAFs) on the build plate are less than or equal to 100mm in their largest dimension, use the standard 20mm QA cube. For this study 5 cubes per run and no other parts will be included.

### Correct placement and orientation:

The cube(s) may be placed anywhere on the build plate, provided the black stripe on the orientation plate is curving along the direction of build plate rotation. In this way, the “Z” label will be facing up, the “Y” will be facing radially outward toward the outer edge of the build plate, and the “X” label will be in the axis of rotation facing the clockwise side.

### Post-processing:

The QA cube should have the supports removed and be post-processed similarly to other models. The orientation plate may be discarded without post-processing.

Make sure to bag and label the cubes after post-processing to avoid confusion with other cubes. Use a permanent marker to label the bag. An example would be “Inner 5” if it is the 5^th^ set of Inner cubes printed. Deliver the cubes to our QA team once you are done.


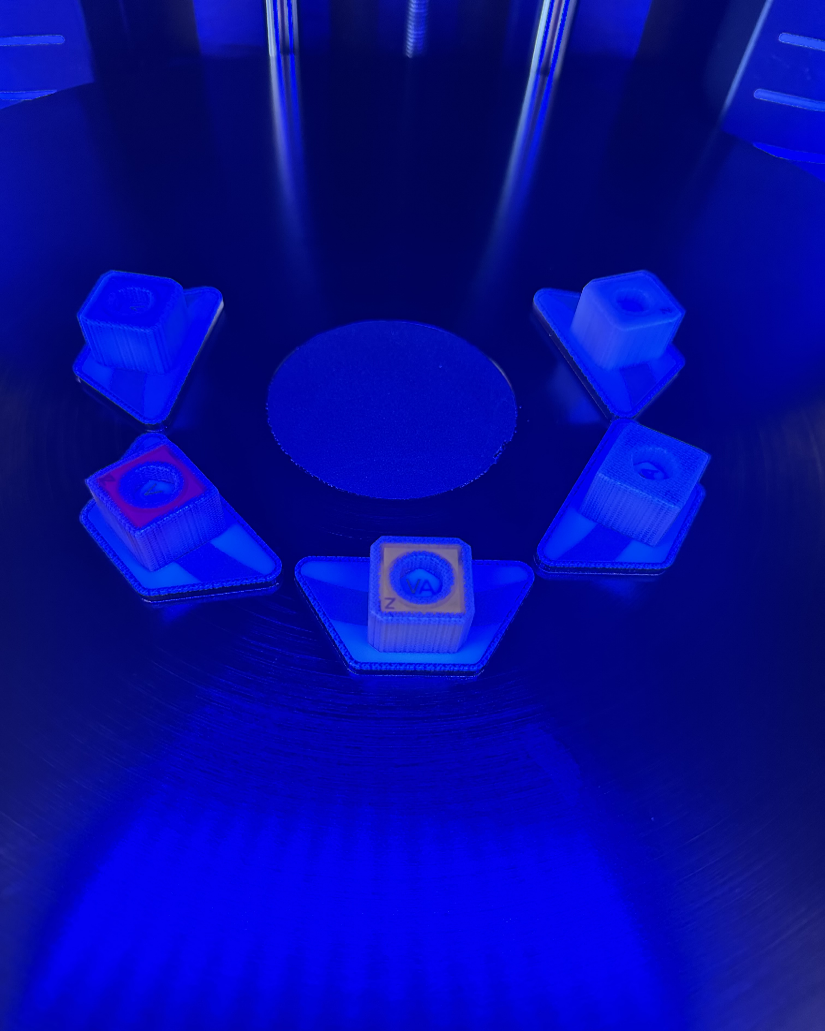


*Figure 5: Finished print of “5 QA Token Inner” with orientation plates facing the center of the tray*


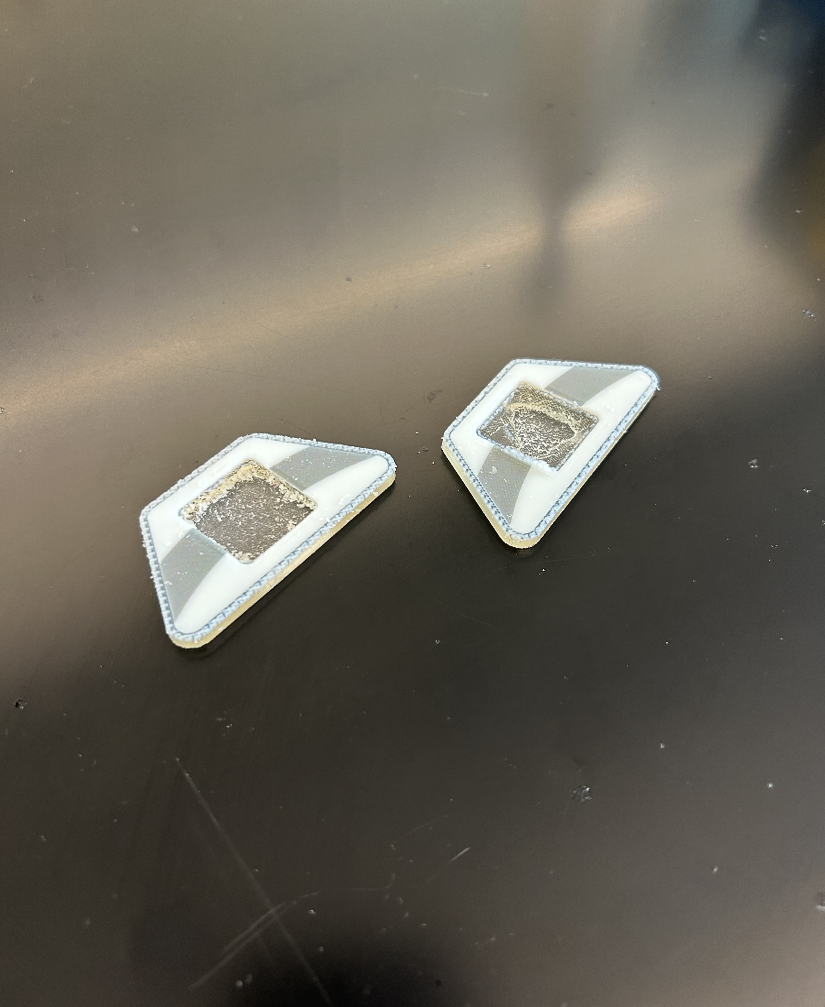


*Figure 6: During post processing, discard the orientation plates once removed from the cubes*

Measurements:

Measure and record

Required tools: Digital calipers with inner- and outer-dimension jaws (or comparable tool)


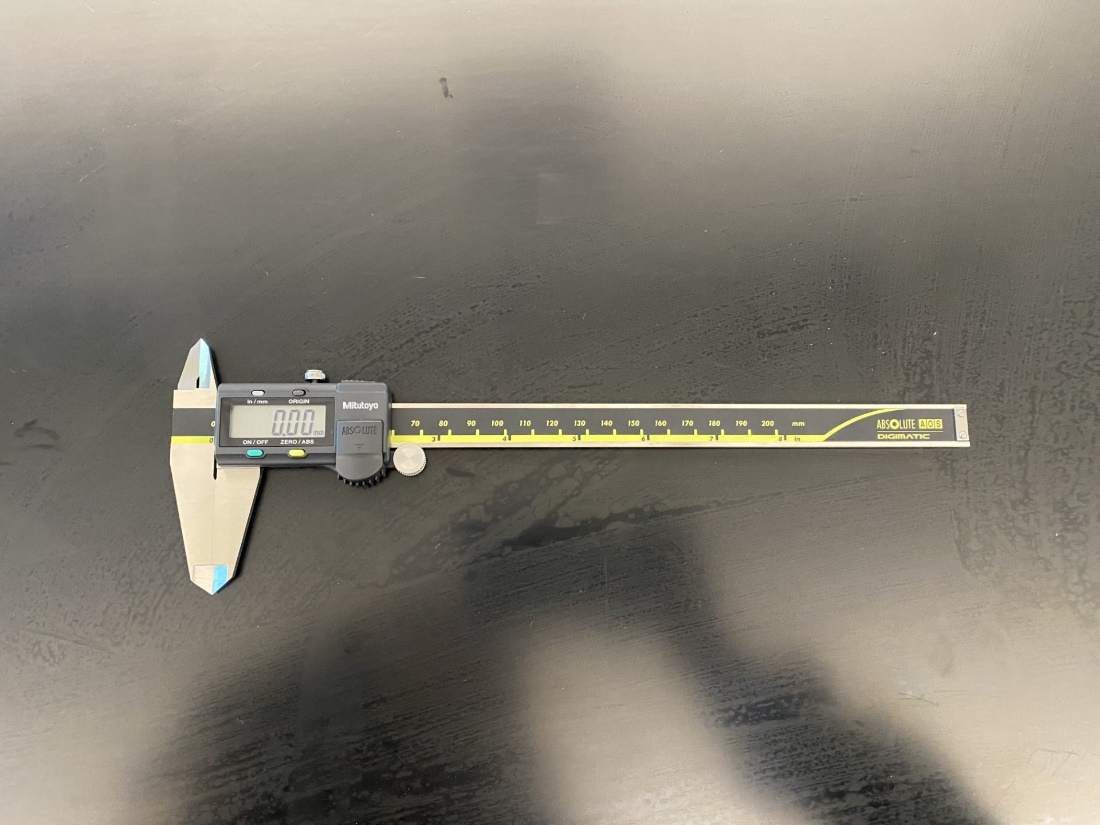


*Figure 5: Digital Calipers Model- Mitutoyo ABSOLUTE CD – 8” AX Serial number: B22356858*

The VA uses a digital protractor (or comparable tool). Note that it can be difficult and time-consuming to obtain a precise measurement of a small object using such a device. It is sufficient to set a digital protractor to 90.0 degrees or use a carpenter/framing square and visually verify that the QA cube fits tightly against it. OSU will not be completing this step. We reference our process below in step 7.

*For both cube sizes, linear tolerances are ±250µm, or 0.25mm. Angular tolerance is 0.3 degrees if precisely measured, but visual confirmation against a known 90.0-degree reference is sufficient for medical models for presurgical planning*.

**Precisely measuring and recording all six linear values is always required.**

**NOTE:** All outer measurements must be measured where the caliper’s prongs are "underlining” the letter (X, Y, or Z depending on face measurement).


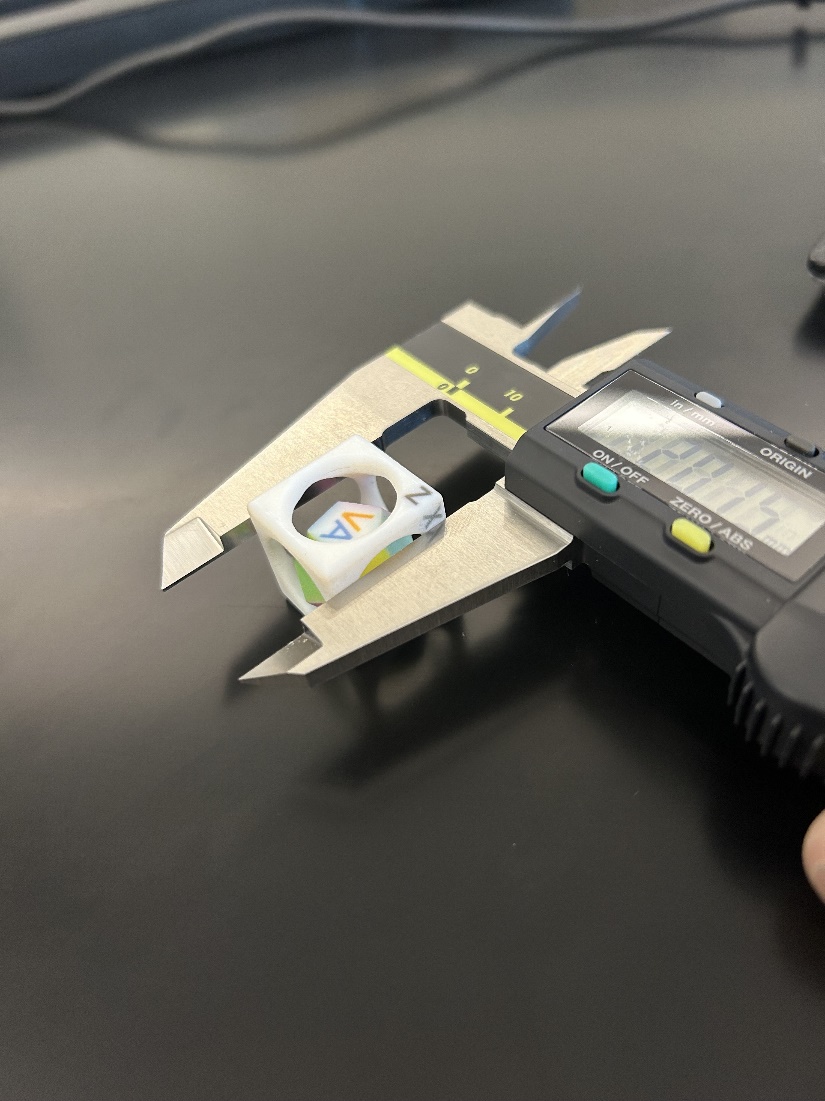


*Figure 7: The caliper must be "underlining” the letter (X, Y, or Z) for the outer measurements- do not place at edge or in middle.*

Outer Dimension Measurement

1. Outer dimension from X face to opposite face


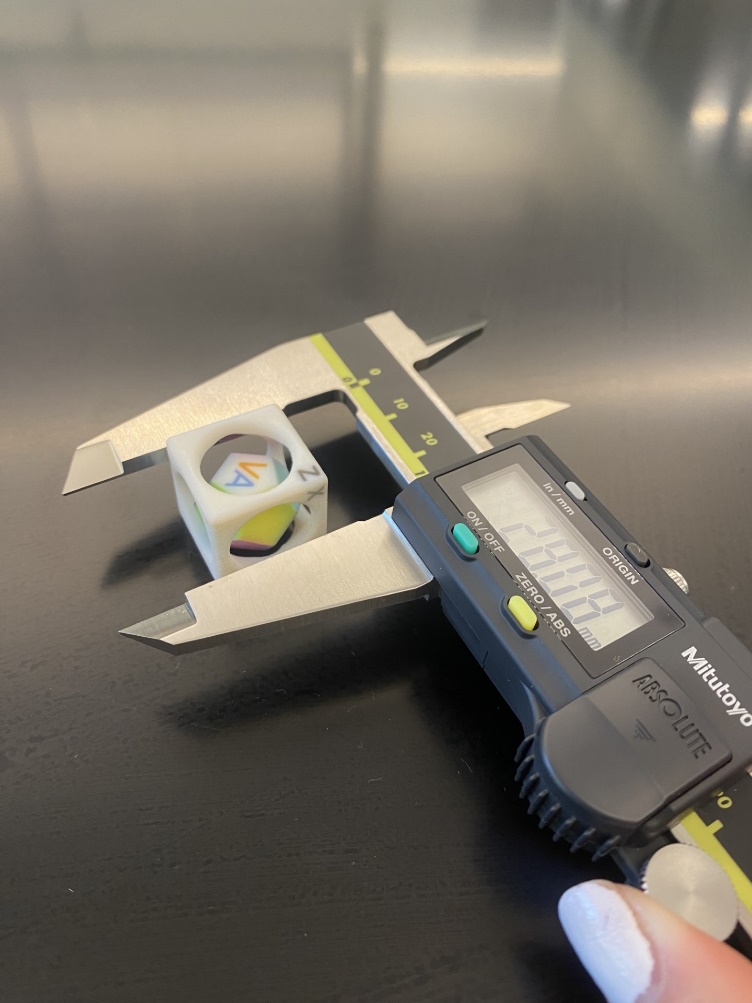


*Figure 8: Opening the caliper and placing the cube to measure the outer X dimension from the X face to the opposite face*


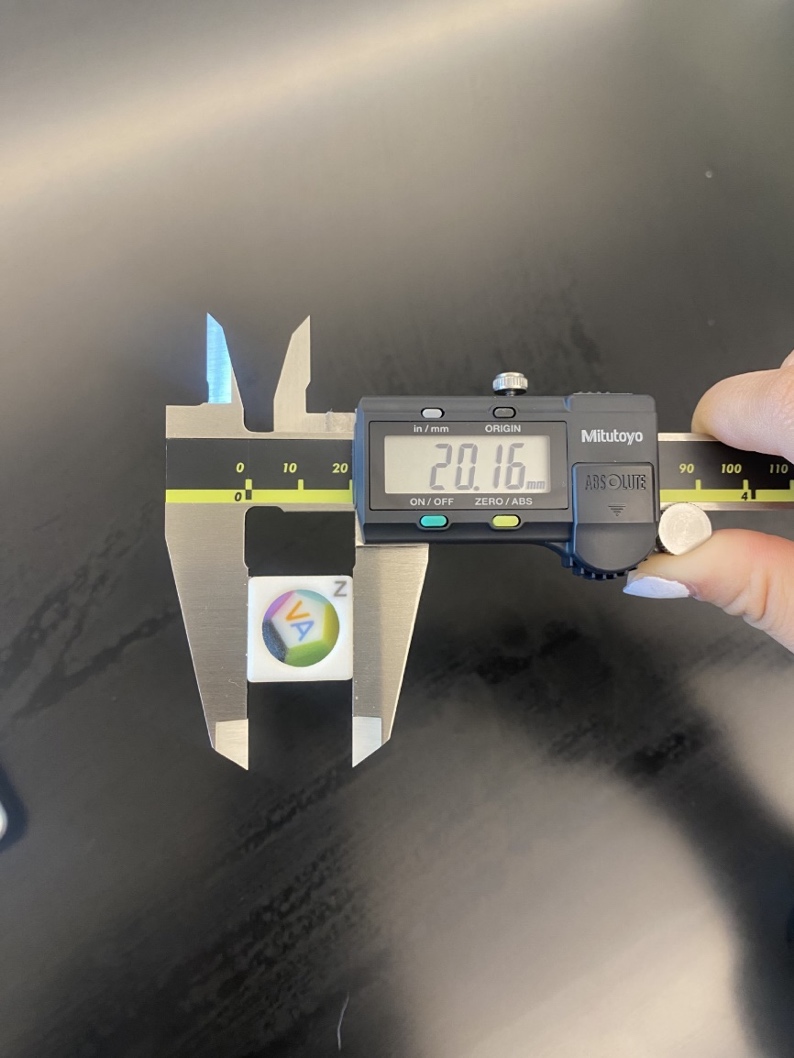


*Figure 9: Measurement of the outer X dimension from the X face to the opposite face*

1. Outer dimension from Y face to opposite face


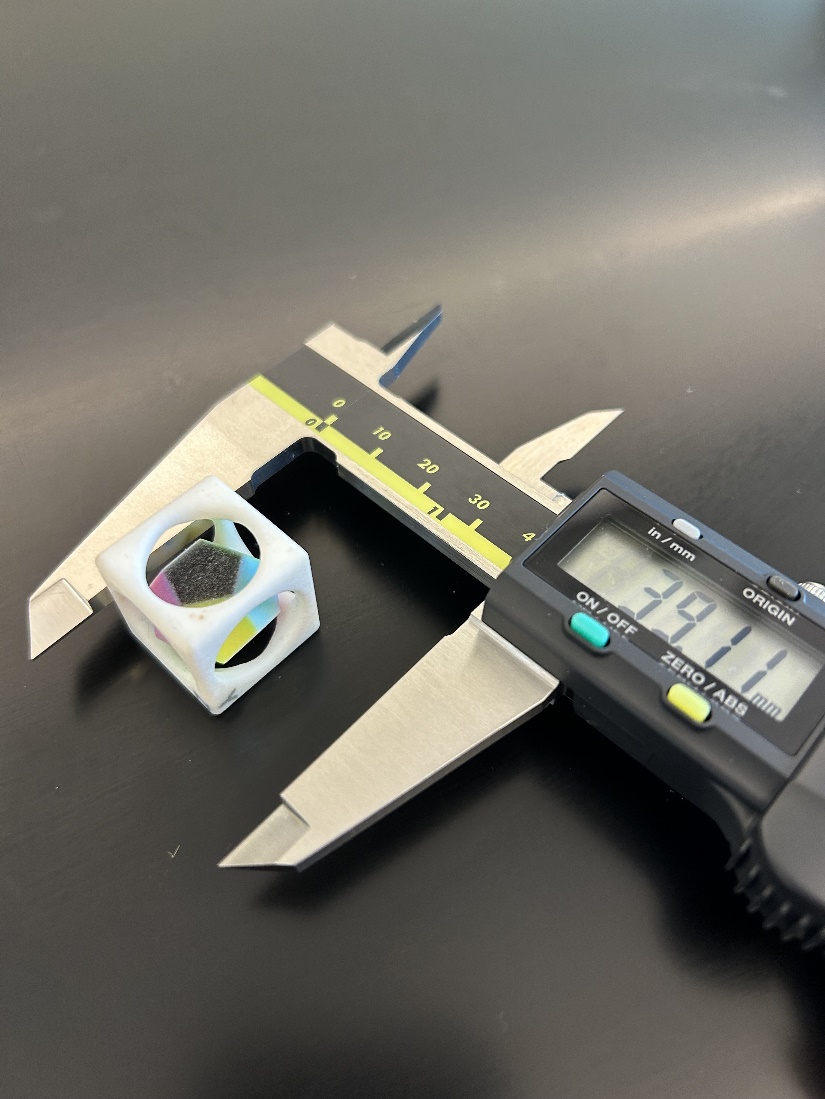


*Figure 10: Opening the caliper and placing the cube to measure the outer Y dimension from the Y face to the opposite face*


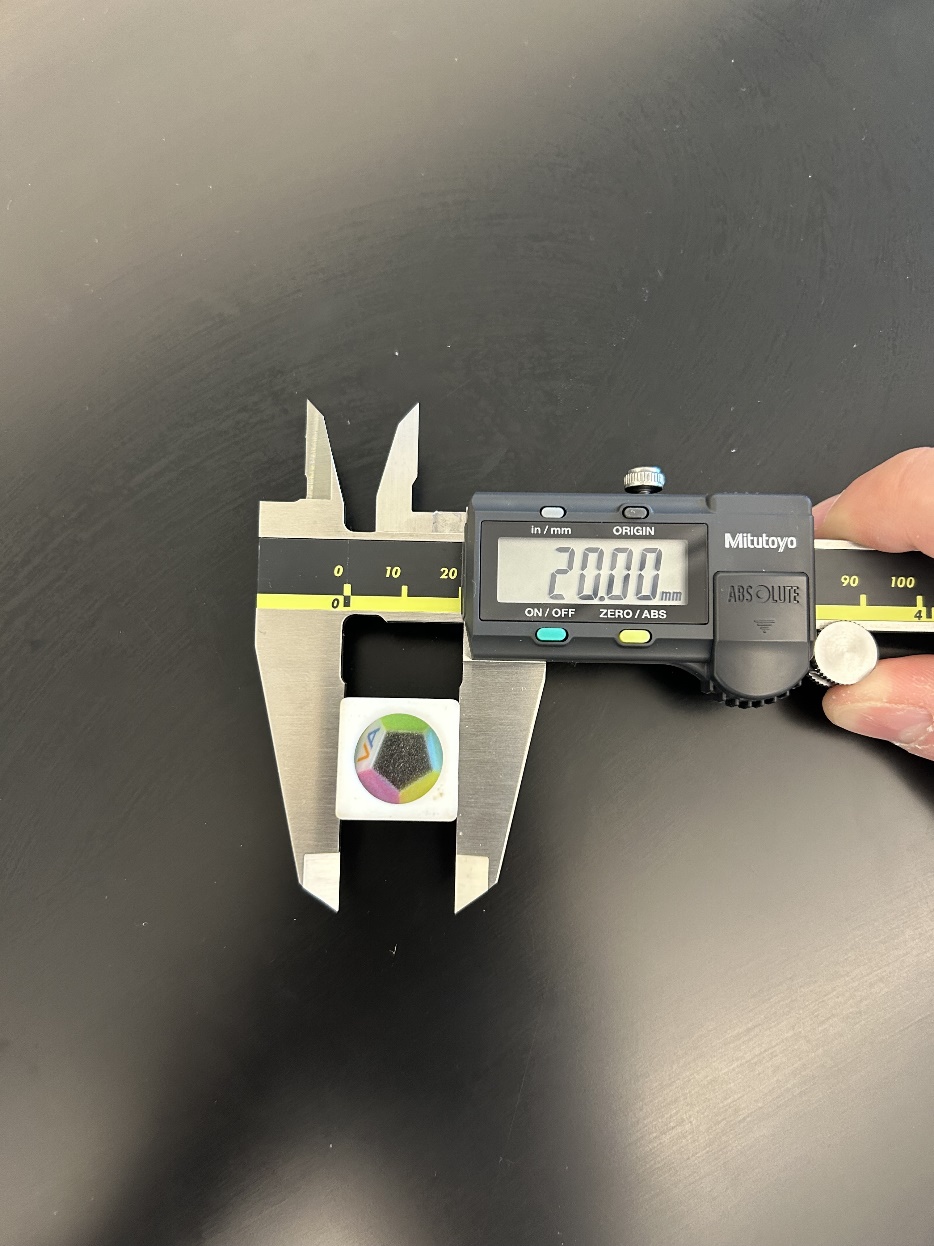


*Figure 11: Measurement of the outer Y dimension from the Y face to the opposite face*

1. Outer dimension from Z face to opposite face


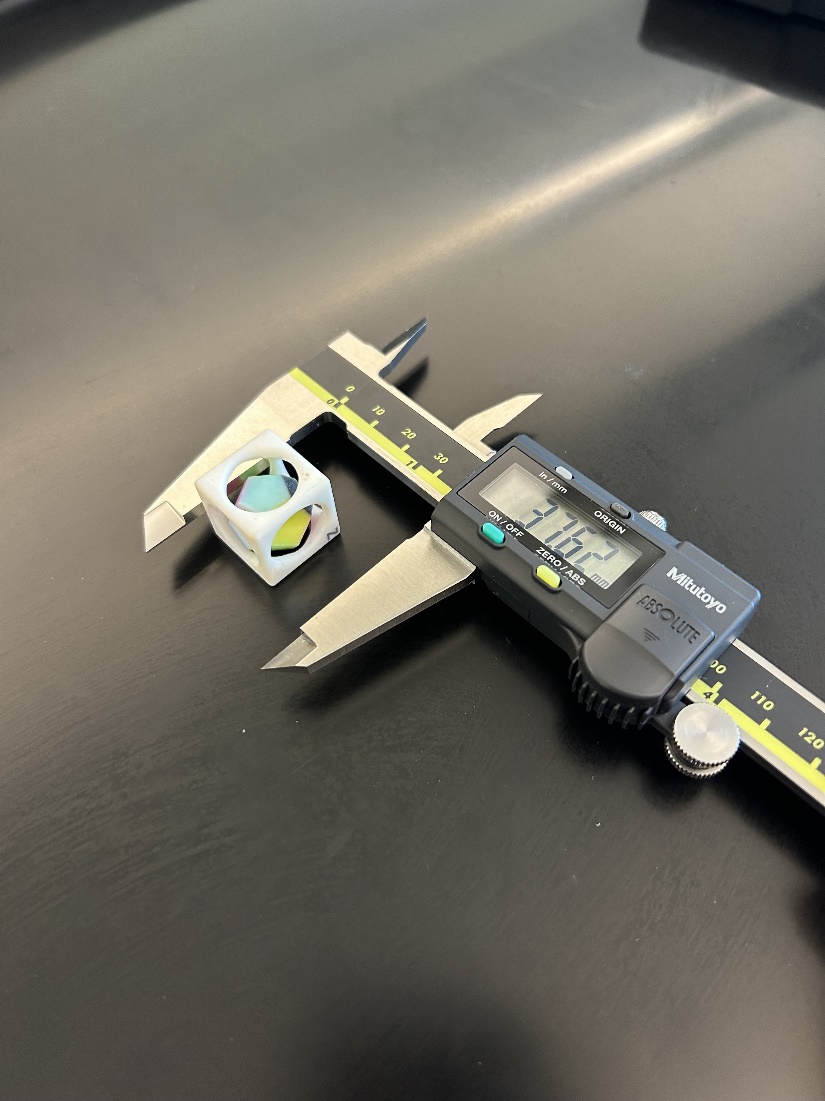


*Figure 12: Opening the caliper and placing the cube to measure the outer Z dimension from the Z face to the opposite face*


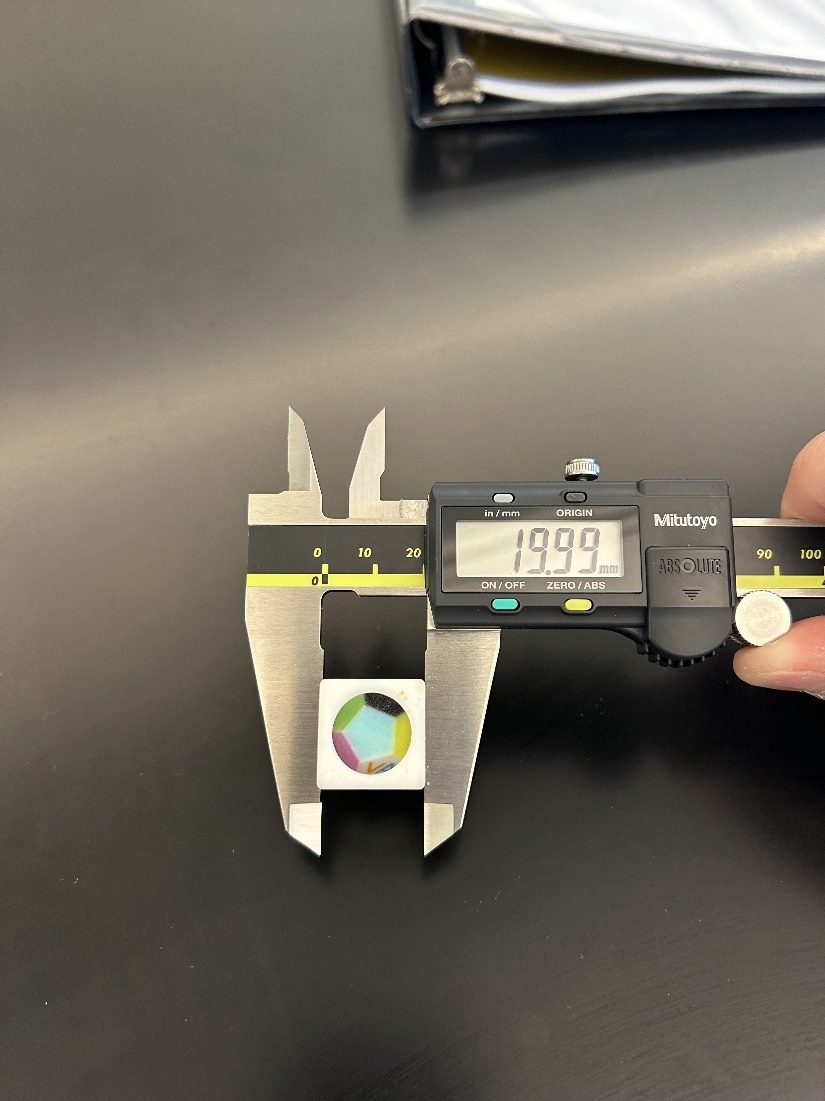


*Figure 13: Measurement of the outer Z dimension from the Z face to the opposite face*

Inner Dimension Measurement

1. Inner dimension in the “X” direction on the Z face.


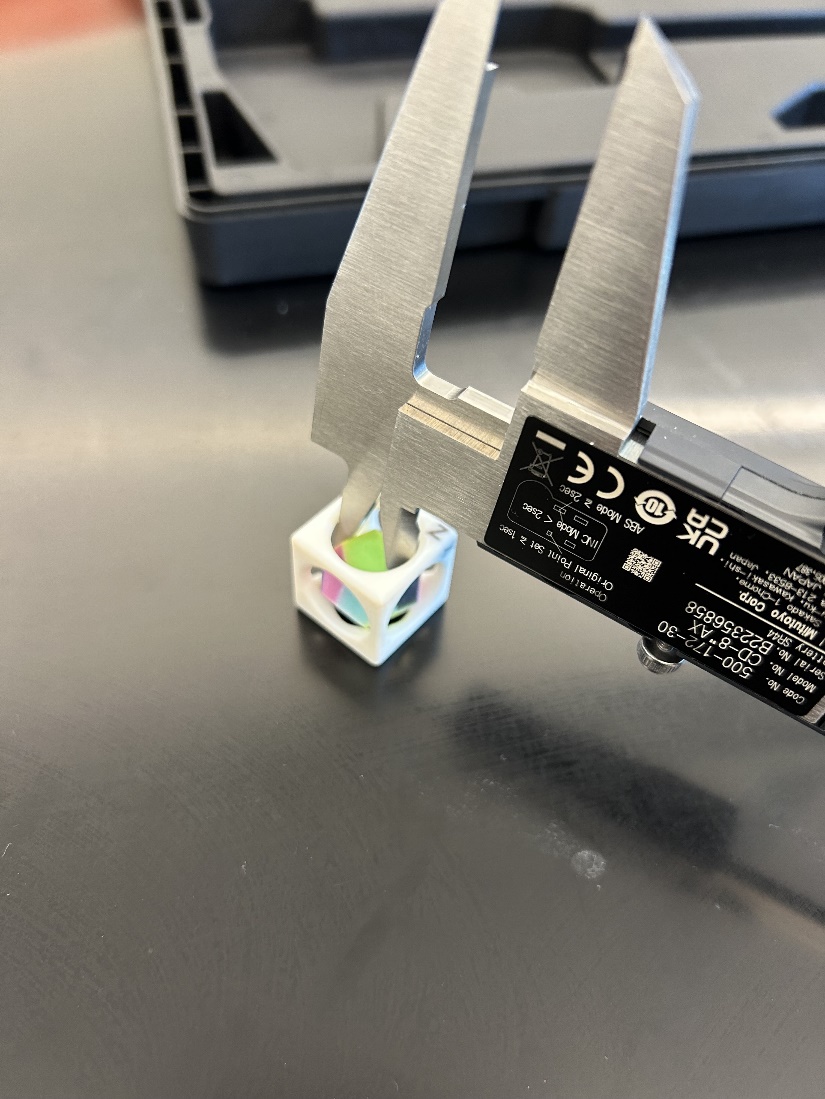


*Figure 14: Opening the caliper and placing the outside prongs inside the Z face of the cube to measure the inner X dimension through the Z face*


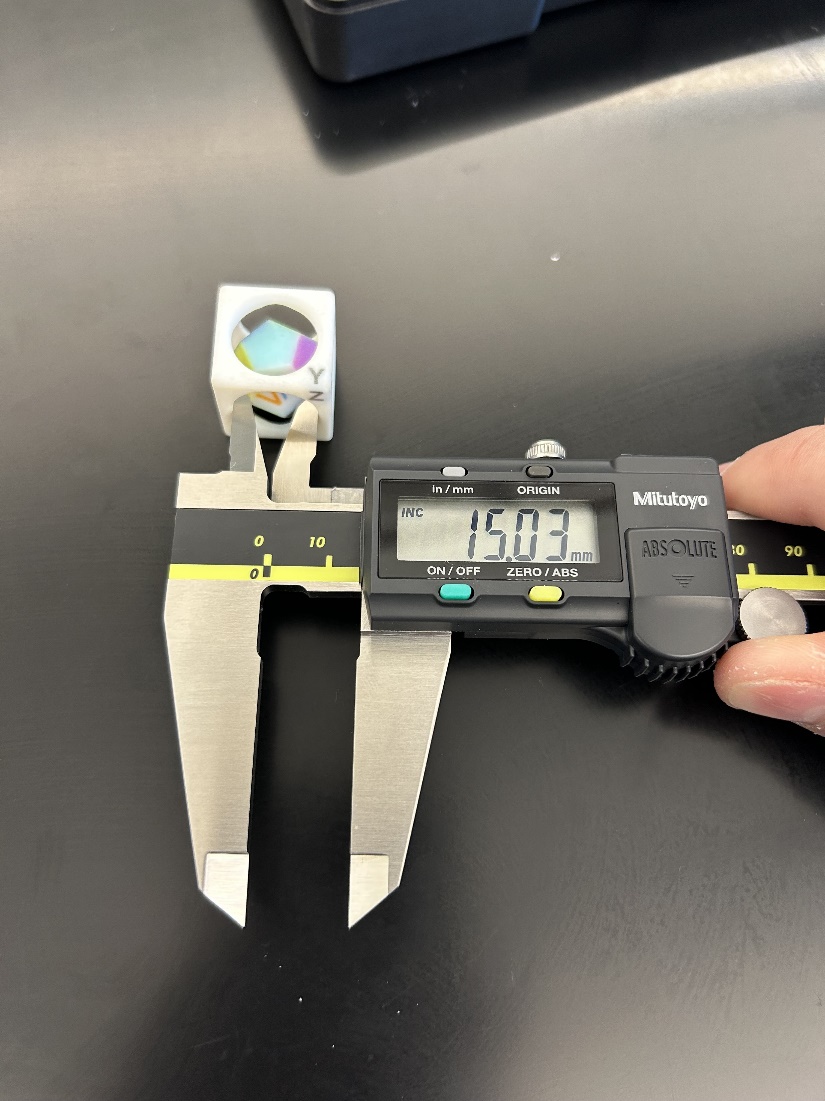


*Figure 15: Measurement of the inner X dimension through the Z face*

1. Inner dimension in the “Y” direction on the X face.


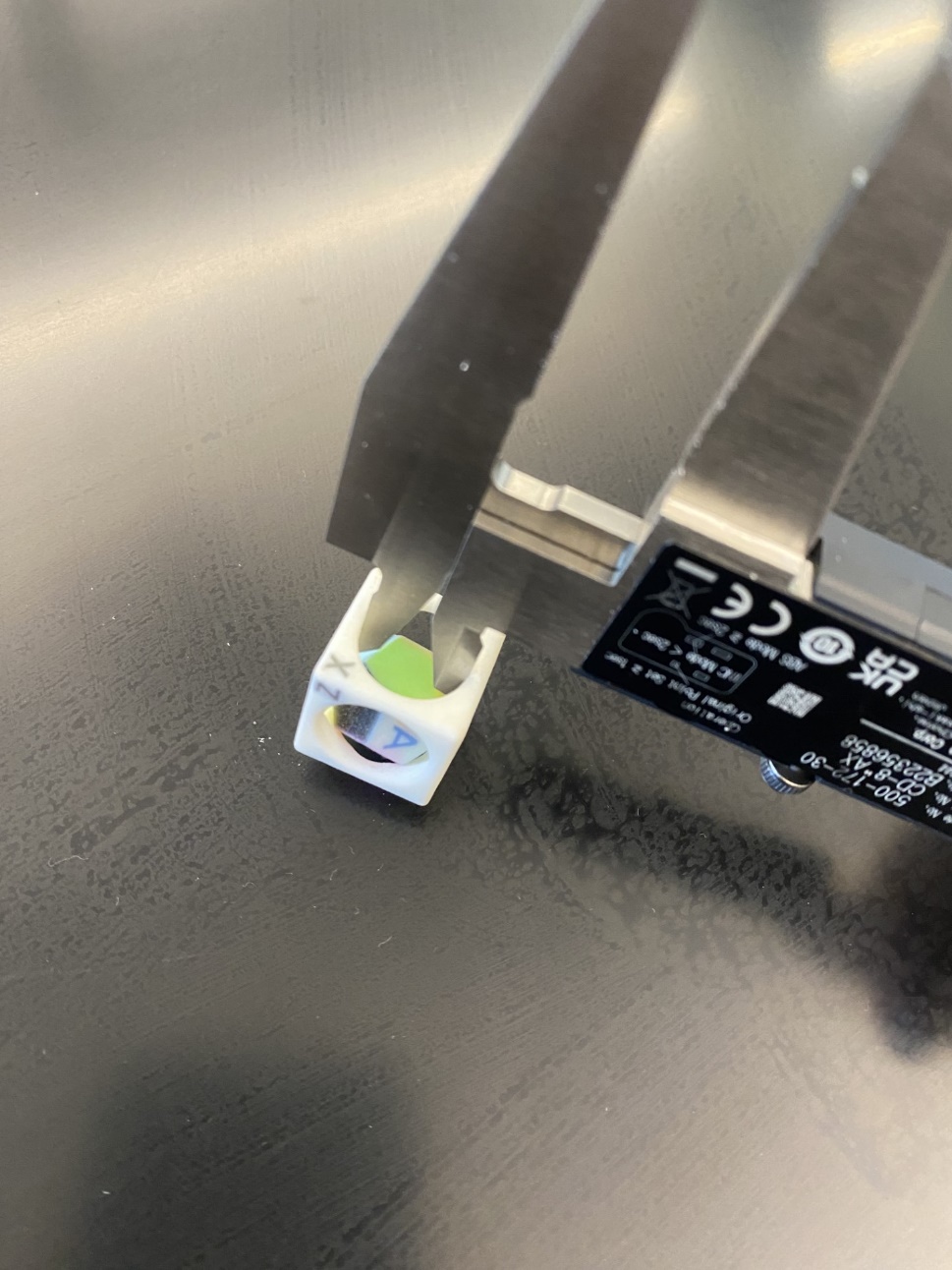


*Figure 16: Opening the caliper and placing the outside prongs inside the X face of the cube to measure the inner Y dimension through the X face*


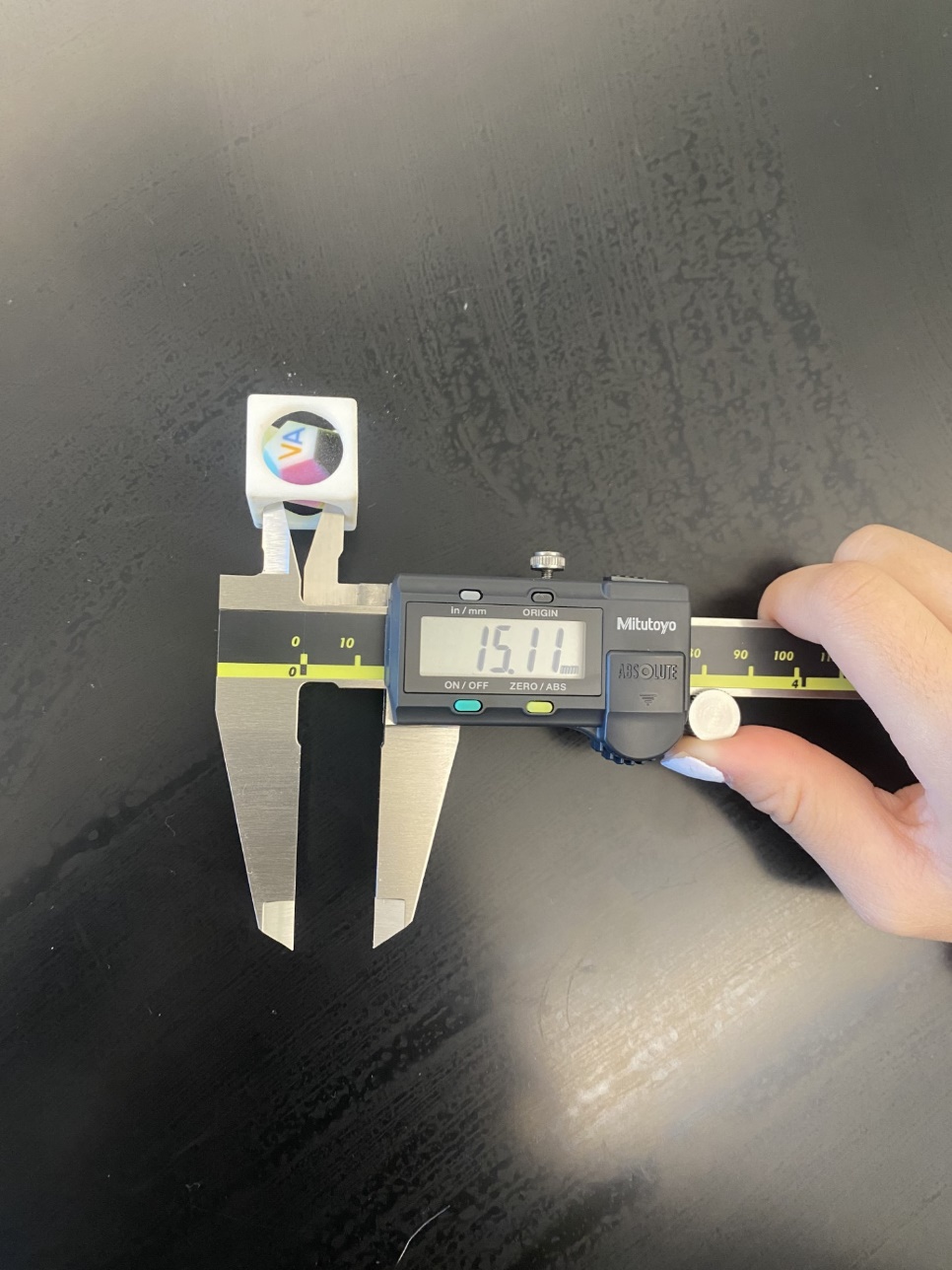


*Figure 17: Measurement of the inner Y dimension through the X face*

1. Inner dimension in the “Z” direction on the X face.


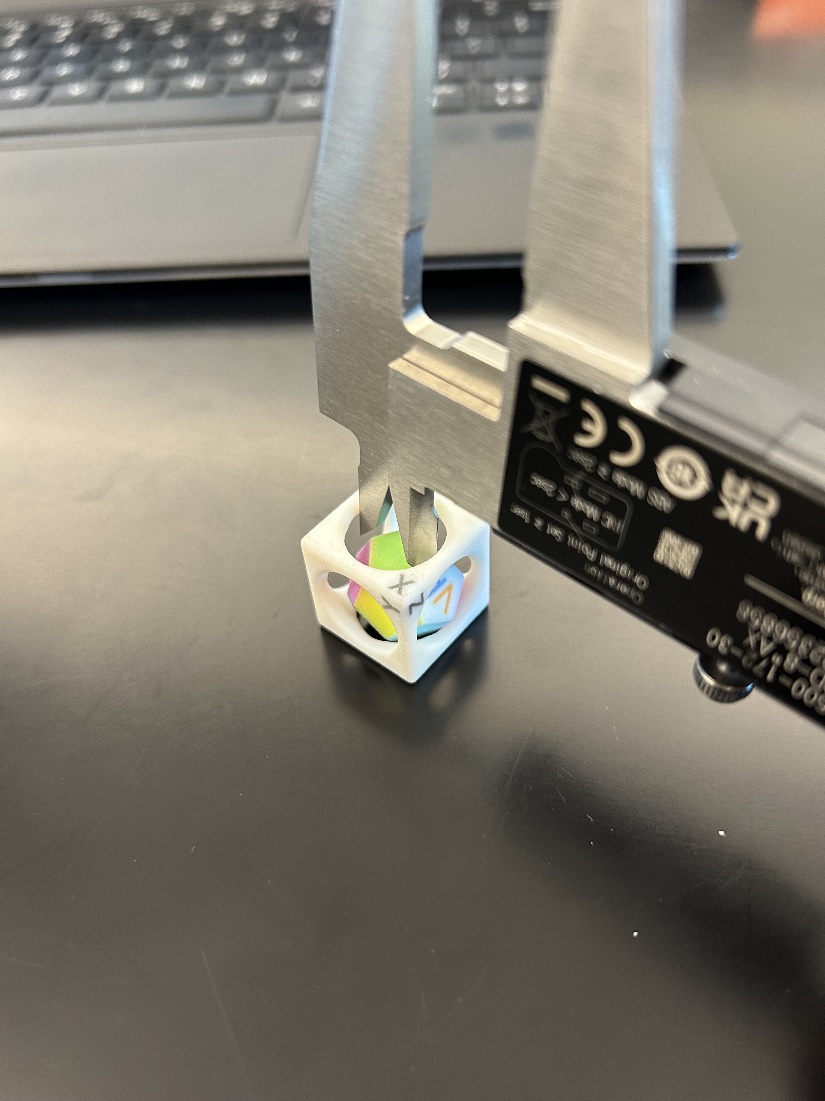


*Figure 18: Opening the caliper and placing the outside prongs inside the X face of the cube to measure the inner Z dimension through the X face*


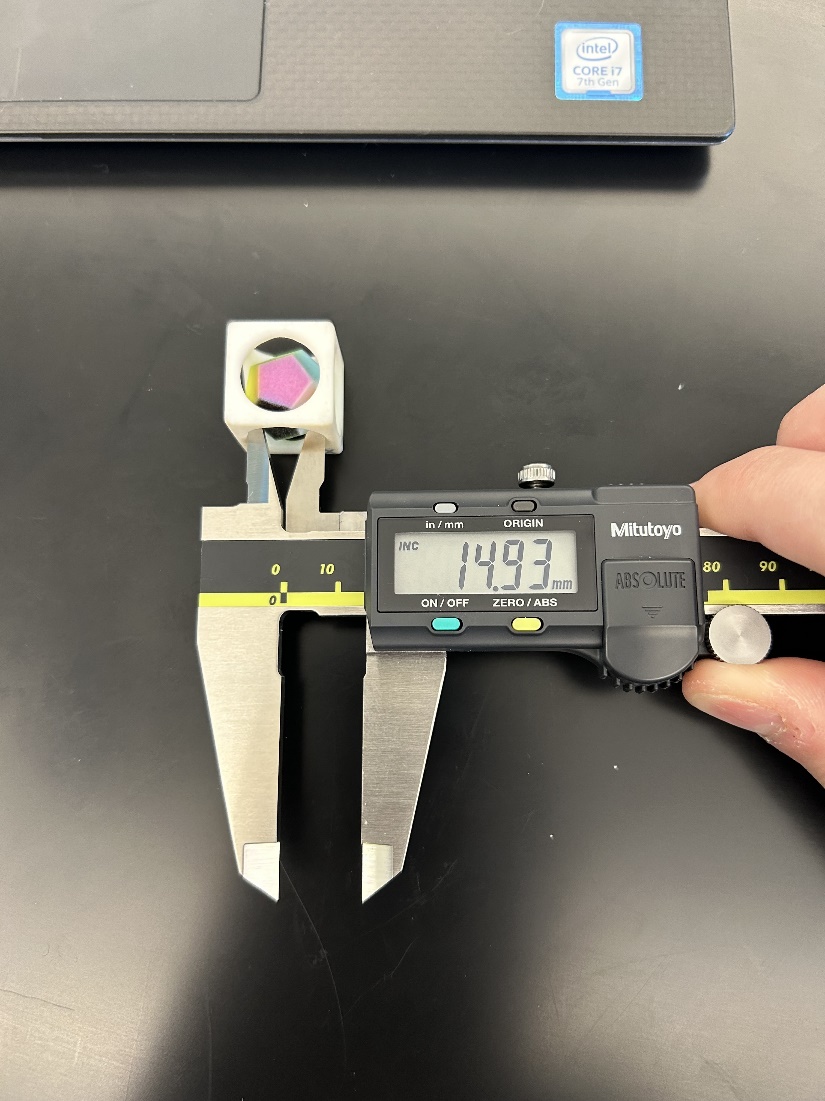


*Figure 19: Measurement of the inner Z dimension through the X face*

*Pass/fail check for Cube square*

1. Verify that the angle between the X/Y, X/Z, and Y/Z faces is 90 degrees by placing the labeled corner inside the digital caliper’s corner. Close the caliper and have the cube tightly fit within the caliper’s jaws. Visually check that the cube is 90 degrees once inside the jaws. This is a pass/fail visual check.


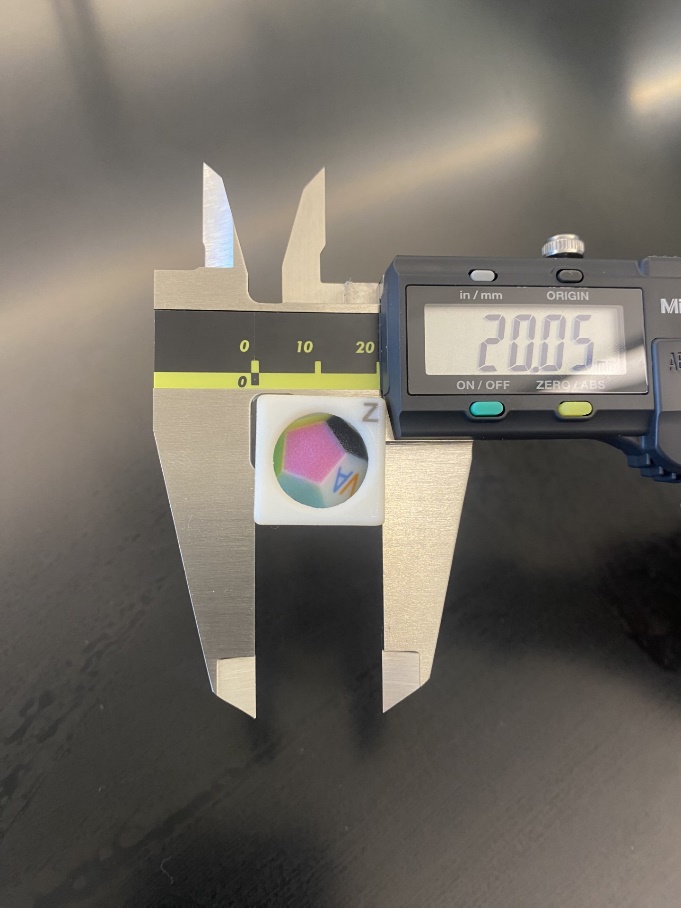


*Figure 20: Closing the cube on the inner corners of the caliper to check for skew*

If any of the measurements are outside tolerance or otherwise suspect, check for thin layers of unremoved support material by gently scraping with the caliper jaws, repeating post-processing and measurements, if necessary, before rejecting the entire build plate.

Recording of measurements

To ensure accuracy, technicians will only be allowed to record 20 cubes at a time.

Practice/Training must be completed prior to measuring and documenting the cubes.

When recording measurements, confirm that the measurements are typed within the correct row and column before typing the measurements. For the “Skew Check” column, there is a drop-down box where the technician will select “Pass” or “Fail” depending on the visual check.

Each resource assigned to measurement will review the work instruction and practice measuring cubes with the lead technician, lab manager, and or quality manager. To reduce potential for errors as a result of fatigue in the measuring activity no more than 30 cubes will be measured within a workday and space out accordingly.

Additional Measurements:

Measure and record

Required tools: Mitutoyo Absolute Stand Gauge


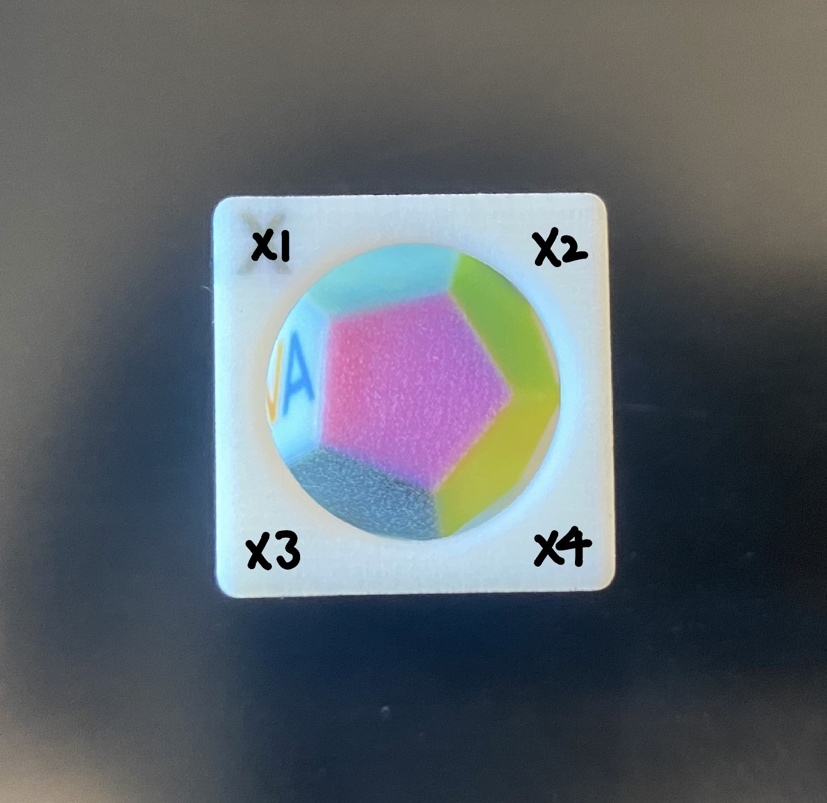


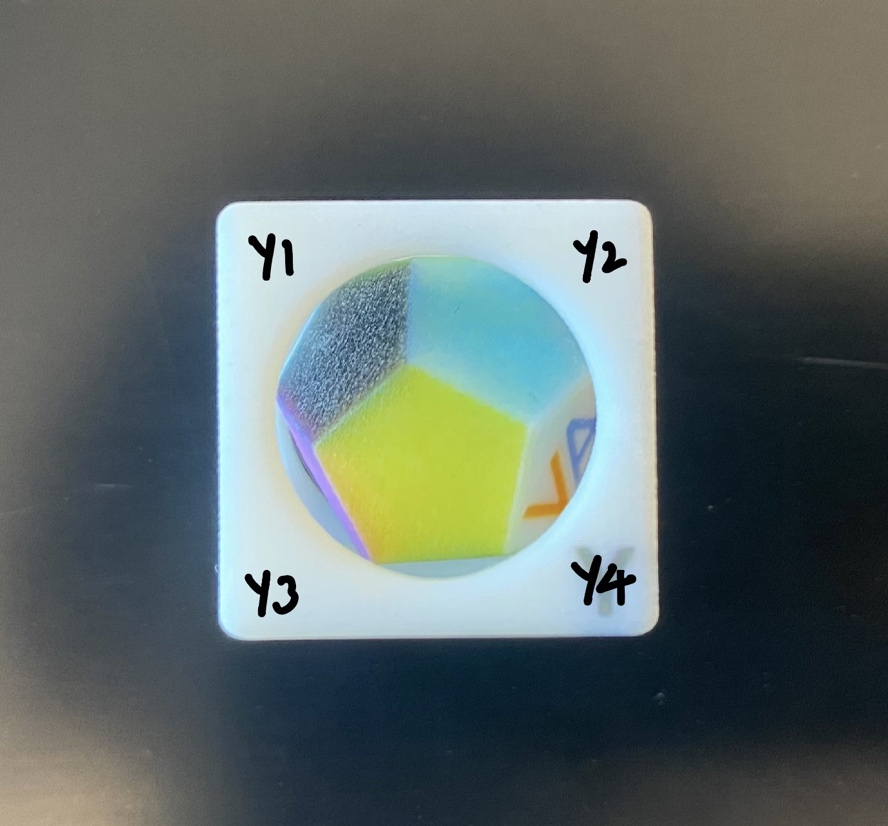


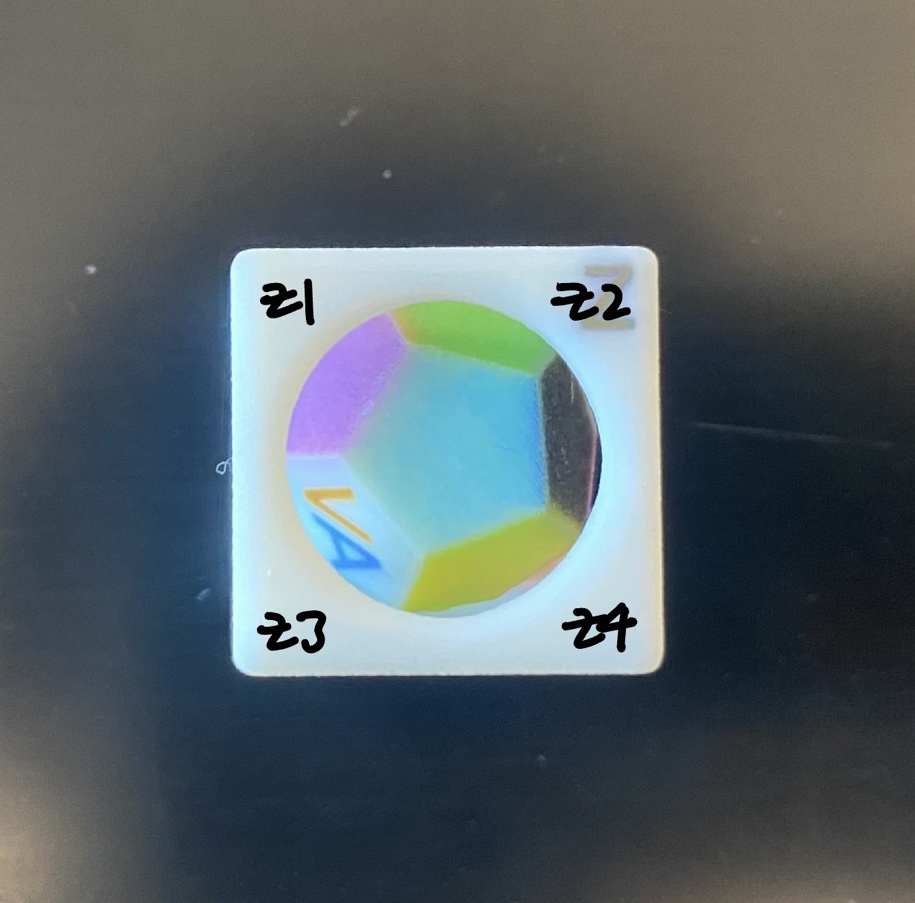

Supplement: Supplementary file 1 — Supplementary Material 1 [file 41205_2025_259_MOESM1_ESM.docx]
