# Supplementary figures and images for: Multi-Site evaluation of a novel point-of-care 3D printing quality assurance protocol for a material jetting 3D printer
Source: 3D Print Med. 2025 Mar 6;11:10. doi: 10.1186/s41205-025-00259-w (PMC11883906; doi:10.1186/s41205-025-00259-w)

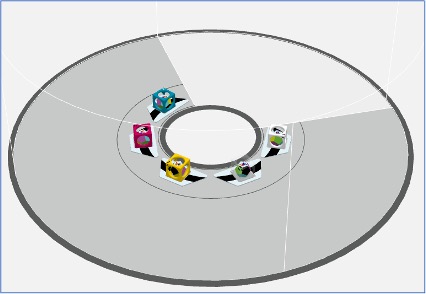

Supplement: Supplementary file 2 — Supplementary Material 2 [file 41205_2025_259_MOESM2_ESM.jpg]
